# Supplementary material for: Effect of Serial Systemic and Intratumoral Injections of Oncolytic ZIKVBR in Mice Bearing Embryonal CNS Tumors
Source: Viruses. 2021 Oct 19;13(10):2103. doi: 10.3390/v13102103 (PMC8541080; doi:10.3390/v13102103)
Supplement: Supplementary file 1 [file viruses-13-02103-s001.zip › viruses-1340569-supplementary.pdf]

Supplementary materials:

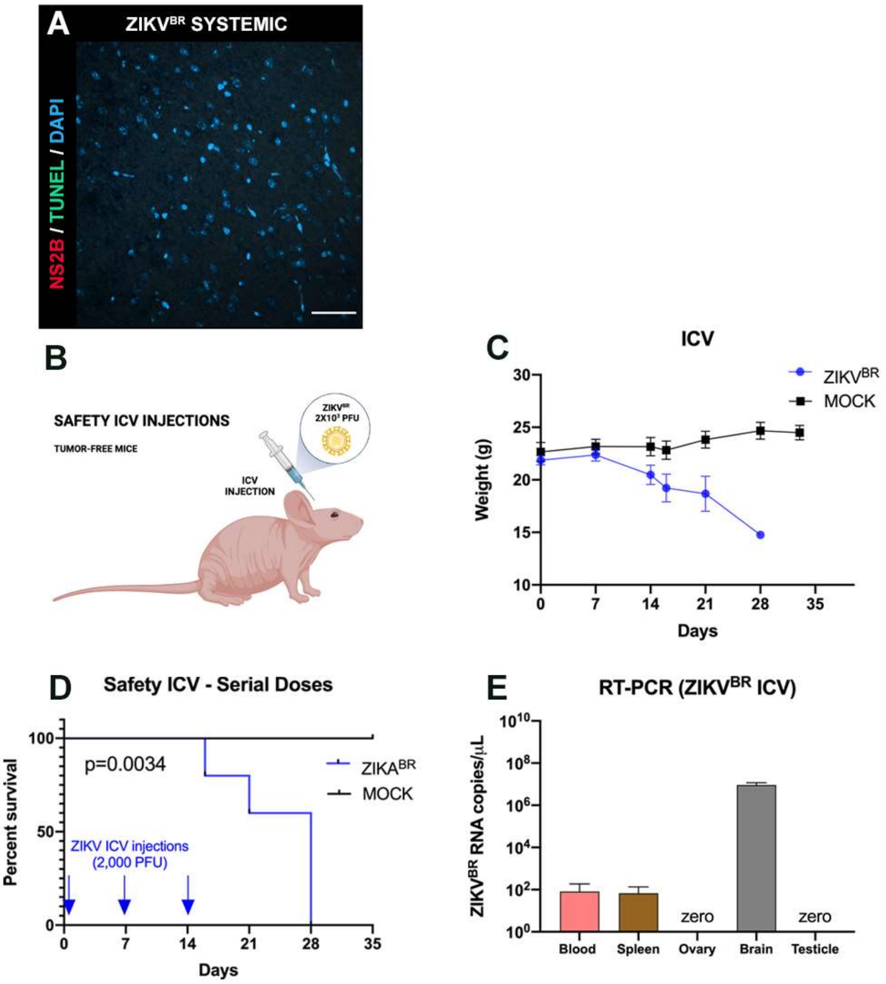

Figure S1: (A) Immunolabeling of ZIKVBR (NS2B, red), cell death (TUNEL, green), and nuclei (DAPI, blue) from cerebral cortex tumor-free mice after systemic ZIKVBR injection. (B) Schematic representation of tumor-free mice treated with ICV ZIKVBR injections. Bodyweight (C), overall survival rate (D), and ZIKVBR RNA were detected by q-PCR (E) in tumor-free mice with three ICV ZIKVBR injections.

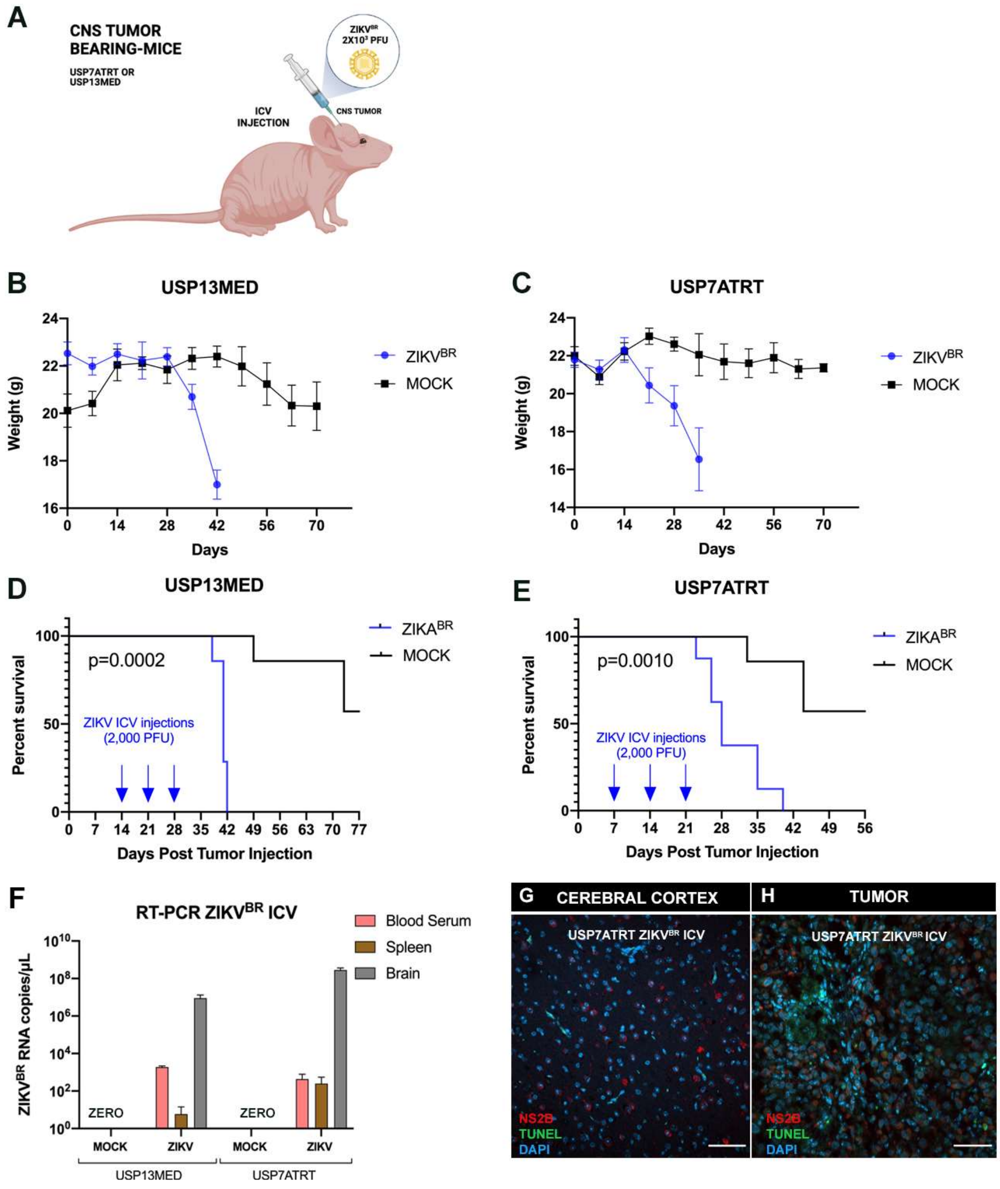

Figure S2: (A) Schematic representation of tumor-bearing mice treated with ICV ZIKVBR injections. Bodyweight (B), overall survival rate (D), and ZIKVBRRNA were detected by q-PCR (F) in USP13MED tumor-bearing mice treated with three ICV ZIKVBR injections. Bodyweight (C), overall survival rate (E), and ZIKVBR RNA were detected by RT-PCR (F) in USP7ATRT tumor-bearing mice treated with three ICV ZIKVBR injections. Immunolabeling positive of ZIKVBR (NS2B, red), cell death (TUNEL, green), and nuclei (DAPI, blue) from the cerebral cortex (G) and tumor (H) of USP13ATRT tumor-bearing mice after ICV ZIKVBR injections.

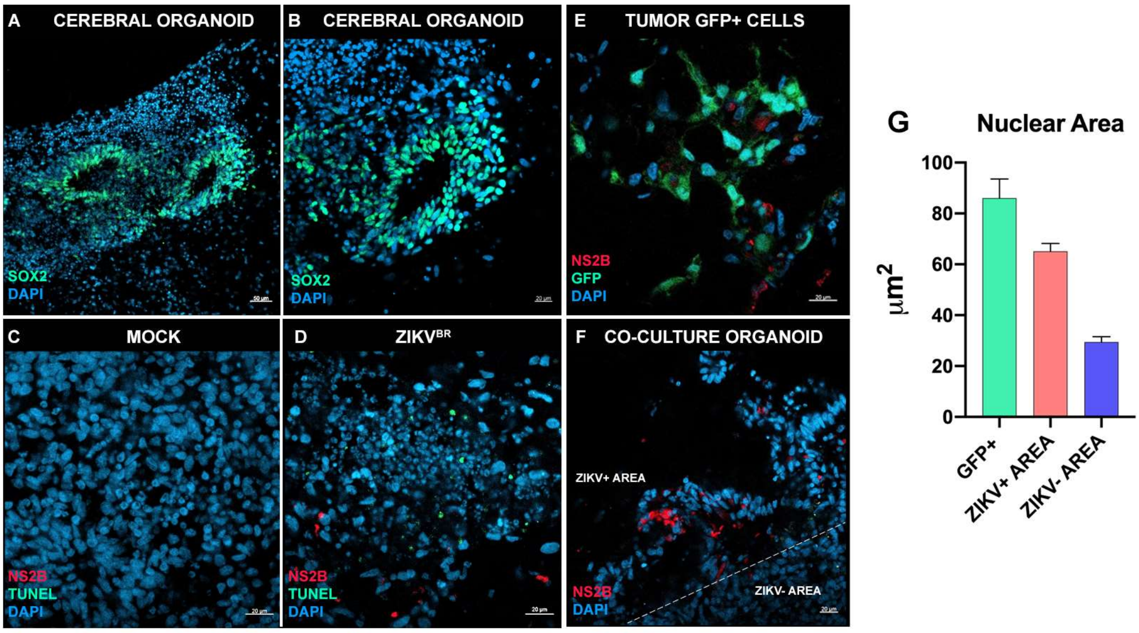

Figure S3: Figure S3: Immunolabeling positive of SOX2 (green) and DAPI (blue) from early-stage human cerebral organoids (A-B) and positive of ZIKV non-structural protein NS2B (red), cell death (TUNEL, green), and DAPI (blue) from organoids without tumor cells MOCK (C) and ZIKVBR infected (D). (E) Embryonal CNS tumor cells GFP+ (green) co-cultured with cerebral organoid and ZIKVBR infected (red). (F) ZIKVBR non-structural protein NS2B positive and non-positive area in the organoid co-cultured with CNS embryonal tumor and (G) nuclear area quantification of tumor GFP+ cells, ZIKV+ and ZIKV- area in the infected organoid co-culture with CNS embryonal tumor. Scale bar (A):0μm; (B-F):20μm.
